# Neural representations of own-voice in the human auditory cortex

**DOI:** 10.1038/s41598-020-80095-6

**Published:** 2021-01-12

**Authors:** Taishi Hosaka, Marino Kimura, Yuko Yotsumoto

**Affiliations:** Department of Life Sciences, The University of Tokyo, Tokyo, Japan

**Keywords:** Neuroscience, Sensory processing

## Abstract

We have a keen sensitivity when it comes to the perception of our own voices. We can detect not only the differences between ourselves and others, but also slight modifications of our own voices. Here, we examined the neural correlates underlying such sensitive perception of one’s own voice. In the experiments, we modified the subjects’ own voices by using five types of filters. The subjects rated the similarity of the presented voices to their own. We compared BOLD (Blood Oxygen Level Dependent) signals between the voices that subjects rated as least similar to their own voice and those they rated as most similar. The contrast revealed that the bilateral superior temporal gyrus exhibited greater activities while listening to the voice least similar to their own voice and lesser activation while listening to the voice most similar to their own. Our results suggest that the superior temporal gyrus is involved in neural sharpening for the own-voice. The lesser degree of activations observed by the voices that were similar to the own-voice indicates that these areas not only respond to the differences between self and others, but also respond to the finer details of own-voices.

## Introduction

Humans have the ability to distinguish themselves from others. This ability is not limited to humans; some animals also exhibit it^1–3^. The animals that can recognize themselves are sometimes categorized as “intelligent” species. They generally have large brains relative to their body weight and show evidence of social behavior such as empathy^1,4–6^. Therefore, self-recognition seems to be an essential ability to live in a society where individuals socially interact. Humans in particular live in the most complex social structure and have the ability to not only discriminate oneself from others but also to recognize themselves. The present study examines the nature of fine self-recognition in humans.

The ability of self-recognition in humans has been investigated in many studies. In behavioral experiments, the subjects were instructed to observe and respond to the stimuli that represented themselves. Pictures of their own faces have been the most frequently used of such stimuli. For instance, one previous study^7^ presented pictures of each subject’s own face and the face of an unfamiliar person to identify the brain regions selectively involved in the recognition of one’s own face. While pictures are easy to use in experiments, we only observe our faces either in photographs or horizontally flipped images in mirrors. In addition, the time we observe our own face is limited. On the other hand, we are exposed to our own voice whenever we speak. Therefore, one’s voice also constitutes a component of “self” and indeed may be a better, more representative example of real-world self-representation. Thus, we employed voice as the stimulus in this study.

While voice may represent the “self” better than or as well as a picture of oneself, there is a technical difficulty in reproducing a voice stimulus that sounds like one’s own-voice, that is, the voice one hears when one speaks. Sound recognized as own-voice travels to the ear through two pathways: an air-conducted pathway and a bone-conducted pathway via the cranial bones^8^. The recorded voice, on the other hand, only contains sounds conducted through the air. This means that the own-voice includes sounds from both bone conduction and air conduction, while the recorded-voice only includes sounds from air conduction^9,10^. Because of these differences, recorded voices are often perceived as strange even though they are recognized as one’s own voice.

Several studies have examined methods to reproduce the own-voice from the recorded-voice by applying filters that emulate bone conduction^11–13^. Based on previous studies of the transfer function for the own-voice, the equalization filter was used to reproduce the own-voice from the recorded voice. Although the filtered voice was rated closer to the own-voice than to the recorded voice, the suggested filter types varied across studies^11–13^. A previous report^14^ compared multiple filters and examined which filter was most effective in emulating the subject’ s own-voice, and found that the best filter differed across subjects, indicating large individual differences, and that perception of the own-voice is constant across different sessions within a subject, indicating the stability of own-voice perception.

Neural mechanisms underlying the perception of self have been examined using faces^7,15–28^, as well as voices^18,29–31^. These studies reported higher neural activities in subjects observing their own faces/voices than others’ faces/voices. The areas exhibiting such activity patterns include the right inferior frontal area^18,23–25,29^, the parietal area^15,20,22^, and the inferior temporal area^18,24,25,28^. The brain regions exhibiting higher activities for own-faces/voices were distributed unilaterally with right hemisphere dominance, but differed across studies, indicating that self-recognition takes place in multiple cortical areas.

Comparing one’s own voice with the presented stimulus and detecting a mismatch requires the ability of self-recognition. A previous research^32^ measured neural activities while the subject spoke in the scanner and heard the feedback. The feedback was either another person’s voice, distorted own-voice, or their own undistorted voice. When neural activation was compared, the bilateral superior and middle temporal gyri showed greater activity with self-distorted feedback and other’s voice feedback than with self-undistorted feedback. Similar results have been reported using voice stimuli^33–35^. However, the focus of these studies was on the interactions between motor and sensory processing systems and their findings were explained based on forward models, which allow for the outcome of any action to be estimated and used before the actual sensory feedback becomes available. When the subject spoke and heard the feedback, the feedback was compared to the predicted outcome, and a mismatch between them triggered a corrective signal^36,37^. In reality, however, we are able to detect the differences between our own voice and the presented voice while passively listening to them without speaking.

The recognition of voices is also related to the ability to identify a person. The bilateral middle and superior temporal gyrus (STG), the bilateral inferior frontal gyrus, and right precuneus are involved in person-identity recognition by a vocal sound^38^. A previous study^39^ investigated the neural basis of voice identity with fMRI by comparing the areas involved in the perception of pre-memorized voices and new voices. Their results showed that BOLD (blood oxygenation level dependent) responses in the bilateral middle and posterior superior temporal sulcus (STS) were suppressed when the subjects heard the pre-memorized voices. They attributed this *reduced* neural response to neural sharpening. Neural sharpening is a pattern of neural activity induced by a stimulus that is more typical within an object space. With neural sharpening, a more typical stimulus elicits reduced neural responses. Neural sharpening can be observed with faces^40^ and voices^41,42^, and is considered to reflect long-lasting cortical plasticity. The neural sharpening account suggests that humans are familiarized with the own-voice over a life-long exposure, and therefore exhibit reduced neural responses to it. On the other hand, a voice that does not sound like their own would show less reduction in the neural responses.

Furthermore, it is worth examining the parametric characteristics of the neural responses. Do BOLD signals gradually change with the magnitude of own-voice-ness? Or are there any specific BOLD responses only induced by own-voice perception? In other words, is own-voice coded parametrically or categorically? Previous studies that compared stimuli of self and others did not answer these questions. The present study aimed to investigate the neural correlates of recognition of the own-voice in finer detail by manipulating the subjects’ own voices.

The neural characteristics examined in this study are two-fold: First, we searched for the brain areas exhibiting lower responses to the voice that sounded most similar to the own-voice, consistent with neural sharpening. Second, we searched for the brain areas that exhibited higher responses to the voice most similar to the own-voice, consistent with previous studies that compared stimuli of the self and others.

**Figure 1:**
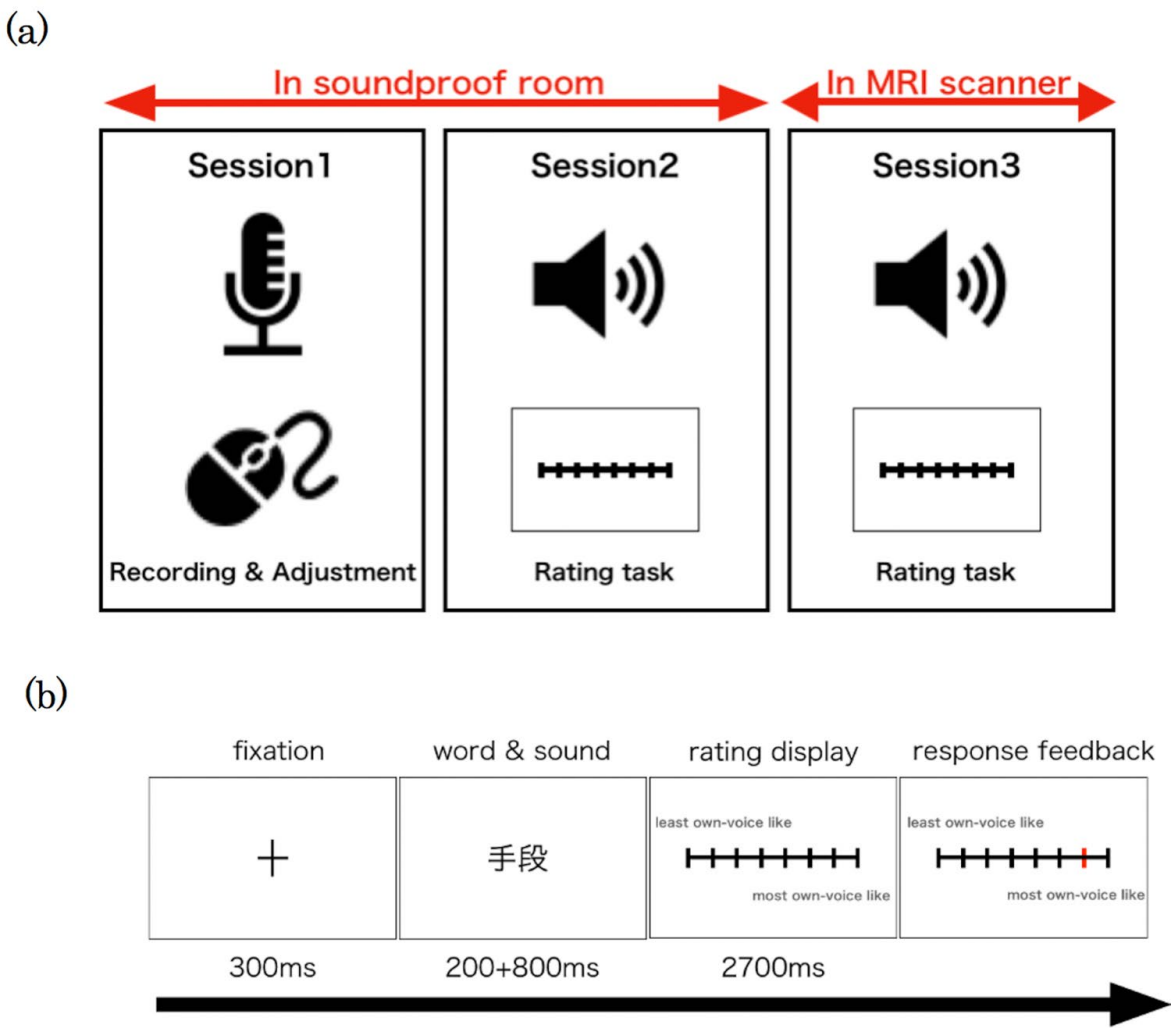
Experimental methods. (**a**) Three sessions were conducted in the experiment. In Session 1, each subject’s voice was recorded and adjusted to reproduce the own-voice. In Session 2, the subjects rated the voice stimuli on an eight-point scale in the soundproof room. In Session 3, the subjects again rated the voice stimuli while the MRI images were acquired. (**b**) Schematic of the task. The subjects rated how much the sound stimuli were like their own voice by pressing the corresponding one of eight buttons. The response was visually shown by changing the color.

The experiment consisted of three sessions conducted on three separate days (Fig. 1a). In Session 1, we recorded the subjects’ voices and applied filters to modify them. We made five types of voices as stimuli (original recorded voice, step filtered voice, bandpass filtered voice, lowpass filtered voice, and adjusted-by-will voice). In the following sessions, each subject rated the five types of voices by how similar they were to their own voice on an eight-point scale ranging from *did not sound like their own voice at all* (1) to *sounded very much like their own voice* (8). We called these ratings the “own-voice score.” The ratings were conducted once in a soundproof room (Session 2) and once during an fMRI scan (Session 3). Because we expected large individual differences in terms of filters that reproduce own voices^14^ and we did not know how the fMRI environment affects our stimuli, we calculated BOLD signal changes separately for each subject based on the behavioral ratings in the fMRI scanner, not on the filter types.

## Results

### Behavioral results

In Session 2 and 3, we categorized the trials based on the responses. The trials with high own-voice scores (7,8) were named as HIGH, and the trials with low own-voice scores (1,2) were named as LOW. In Session 2 (rating task in the soundproof room), the total number of trials across subjects was 335 for LOW trials and 383 for HIGH trials. In Session 3 (rating task in the MRI scanner), the total number of trials across subjects was 1516 for LOW trials and 1461 for HIGH trials. All subjects were aware that the stimuli were generated from their recorded voice, and that there were variations in the stimuli. We first examined consistencies in the own-voice evaluations between the soundproof room and fMRI scanner. For each subject, we specified the filters that earned the highest and lowest own-voice scores. The scores for Session 2 (soundproof room) and Session 3 (fMRI) were compared as a measure of consistency. Of the 17 subjects, 6 showed consistency in filters for both the highest and the lowest scores. Three subjects were consistent only in the filter for the highest scores, and another three subjects were consistent only in the filter for the lowest scores. Five subjects showed no consistency in their ratings.

The voice rated as most similar to own-voice differed across subjects. In the soundproof room, 6 subjects chose the raw recorded voice as most representative of their own voice, while 11 rated modified voices as most like their own voice. In the fMRI scanner, 3 subjects chose the raw recorded voice as most representative of their own voice, while 14 rated the modified voices as most like their own voice. Individual differences were found in the own-voice scores in both environments, indicating that there was no general filter that could reproduce own-voice, as we previously reported^14^. Even though each subject tried to reproduce their own voice by adjusting the pitch, vibrato, and frequency cut-off filter, only a few subjects rated the adjusted voice as the one most similar to their own voice.

**Figure 2:**
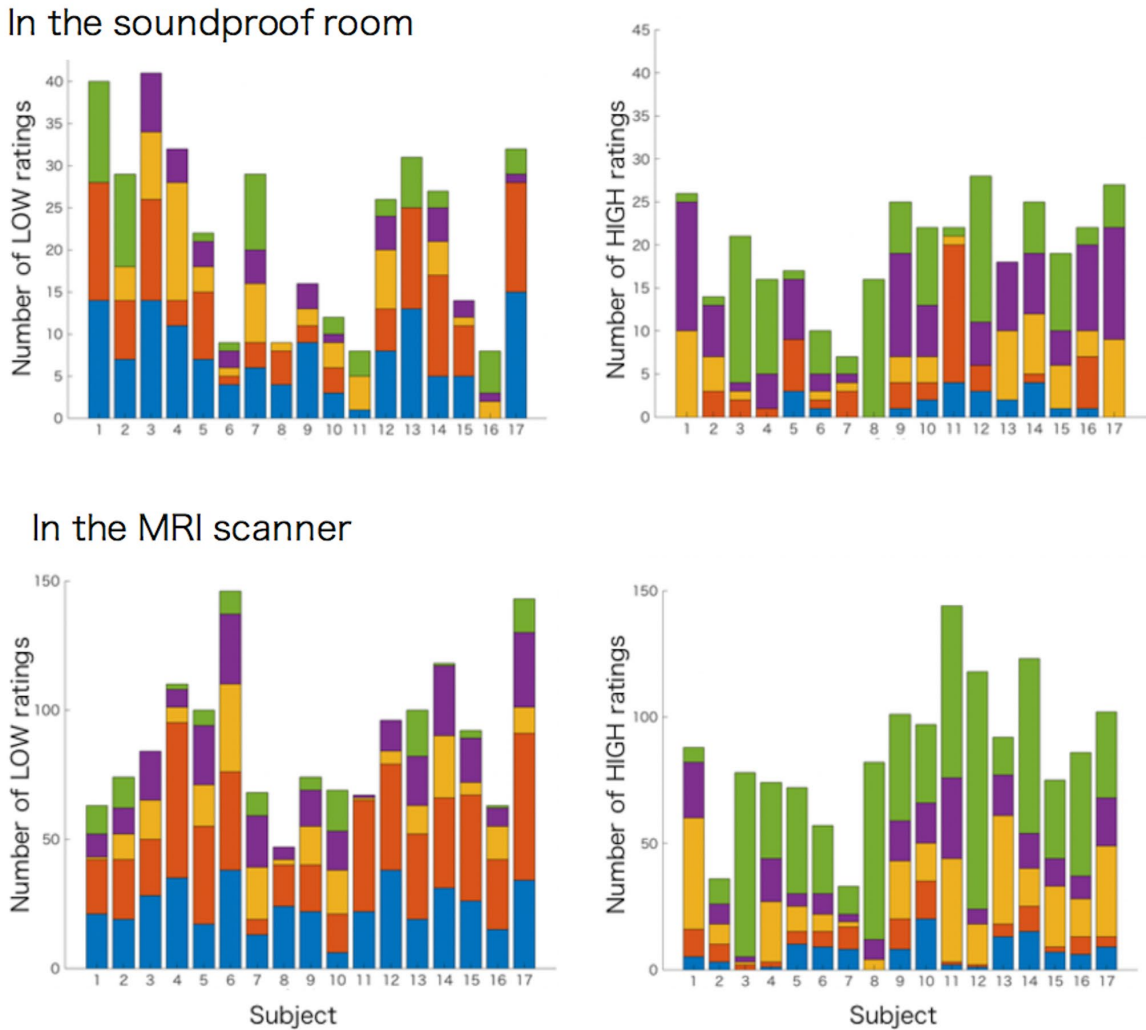
The numbers of LOW and HIGH ratings for each filter condition for each subject. [Upper panel] Rating results in the soundproof room. Left: The number of low-own-voice score (1,2) trials. Right: The number of high own-voice score (7,8) trials; [Lower panel] The rating results in the MRI scanner. Left: The number of low-own-voice score (1,2) trials. Right: The number of high own-voice score (7,8) trials.

The numbers of LOW and HIGH trials for each filter condition for each subject are shown in Fig. 2. The filters with low and high own-voice scores varied across subjects. Figure 2 also shows that both the LOW and HIGH trials included more than one type of filter, indicating that the effects of filters have large between-subject variability and some within-subject variability. Thus, we analyzed fMRI data based on the subjects’ responses, not on the filter types.

### Brain imaging results

We first compared task-related conditions and baseline fixation conditions. The task-related activations were found in the occipital area, temporal area, precentral gyrus, postcentral gyrus, and cerebellum (*t*(16) = 21.77, uncorrected *p* < 0.001, and corrected *p* < 0.05, Table 1).

**Table 1:** The task-related activations.

Cluster size	x, y, z	t	Region
20,562	18, − 79, − 18	21.77	R Cerebelum(VI)
	24, − 52, − 26	19.48	R Cerebelum(VI)
	− 24, − 55, − 22	18.83	L Cerebelum(VI)
	− 6, − 1, 58	17.18	L posterior-medial frontal
	12, − 70, − 22	17.08	R Cerebelum(VI)
	− 66, − 25, 6	16.86	L superior temporal gyrus

Contrasts were significant at a voxel level threshold of p < .001 combined with an FWE correction of p < .05 for cluster size. MNI coordinates and t-scores are from the peak voxel of a cluster (listed 6 peaks).

### Rating effects

To identify regions activated more for voices dissimilar to own-voice than those similar to own-voice, the contrast was calculated by subtracting the activation during trials with high own-voice scores (7,8) from the trials with low own-voice scores (1,2), named LOW–HIGH. We also set the opposite contrast and named it HIGH–LOW.

**Figure 3:**
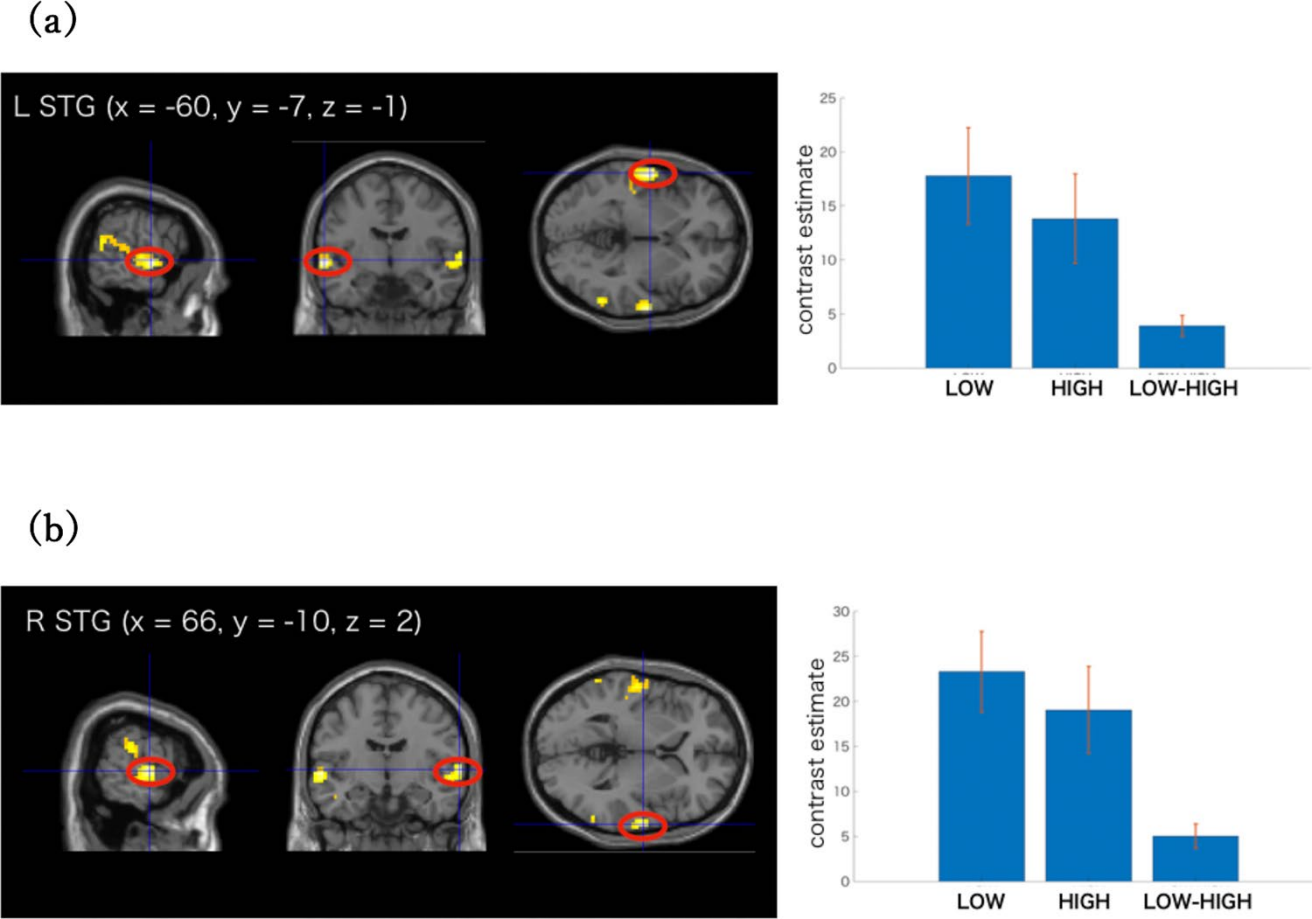
LOW–HIGH contrast. (**a**) Left: Significant voxels for the LOW–HIGH contrast. Right: contrast estimates for LOW, HIGH, and LOW – HIGH in the left STG. (**b**) Left: Significant voxels for the LOW–HIGH contrast. Right: Contrast estimates for LOW, HIGH, and LOW–HIGH in the right STG. Displayed contrasts were significant at a voxel-level threshold of *p* < .001 combined with an FWE correction of *p* < .05 for cluster size. The error bars represent 90% confidence intervals.

The LOW–HIGH contrast revealed that mainly the bilateral temporal area had a higher activation for trials with low own-voice scores than those with high own-voice scores. One significant cluster was found in the left hemisphere and included the middle temporal gyri and superior temporal gyri. In the right hemisphere, two significant clusters were found within the area from the superior temporal gyri to the middle temporal gyri (Table 2). Figure 3a,b show the activation maps of these clusters and the contrast estimates. Both the LOW and HIGH trials exhibited increased activities compared to the fixation trials, but the activations were significantly higher in the LOW trials than in the HIGH trials.

**Table 2:** Rating effects. Results of LOW – HIGH contrast and HIGH – LOW contrast.

Cluster size	*x, y, z*	*t*	Region
**LOW–HIGH**			
218	− 60, − 7, − 1	6.59	L Superior Temporal Gyrus
	− 63, − 52, 16	4.9	L Middle Temporal Gyrus
	− 48, − 25, 10	4.67	L Middle Temporal Gyrus
88	66, − 10, 2	6.13	R Superior Temporal Gyrus
	57, − 10, − 4	5.12	R Superior Temporal Gyrus
	60, 5, − 18	4.96	R Medial Temporal Pole
74	51, − 31, 10	6.65	R Superior Temporal Gyrus
**HIGH–LOW**			
27	21, 35, 41	4.93	R Superior Frontal Gyrus *
12	18, − 64, − 36	4.06	R Dorsal Dentate Nucleus *

Contrasts were significant at a voxel-level threshold of *p* < .001 combined with an FWE correction of *p* < .05 for cluster size. MNI coordinates and *t*-scores are from the peak voxels of a cluster.

* Contrasts were significant at a peak voxel threshold of *p* < .005 (uncorrected), with no cluster thresholding.

No significant clusters were found with the HIGH–LOW contrast. Although not significantly so, the right superior frontal gyri and right dorsal dentate nucleus showed stronger activations for the HIGH trials than the LOW trials (uncorrected *p* < 0.005 and cluster extent > 10 voxels; Table 2).

Next, we examined parametric effects in areas that showed significant differences for the LOW–HIGH contrast. This analysis was conducted to model a linear relationship between BOLD responses and own-voice scores. We set the own-voice score as a factor. The eight rating scores were independently modeled by their own column in the design matrix and were represented numerically from 1 to 8.

We found no significant clusters exhibiting parametric changes corresponding to the eight rating responses. However, with a relatively lenient statistical threshold (*p* < 0.005, uncorrected with at least 10 contiguous voxels; Table 2), regions in the bilateral superior temporal gyrus were found to increase in activity as the own-voice score decreased.

### Effects of filter type

We also examined the effects of filter type on BOLD responses. Data were analyzed using a random-effect procedure. The first stage identified subject-specific activations in all subjects with a design matrix consisting of five filter types and FIXATION as an implicit baseline. To directly assess differences between the filter types, in the second-level group analysis, a one-way ANOVA was performed on the filter types.

**Figure 4:**
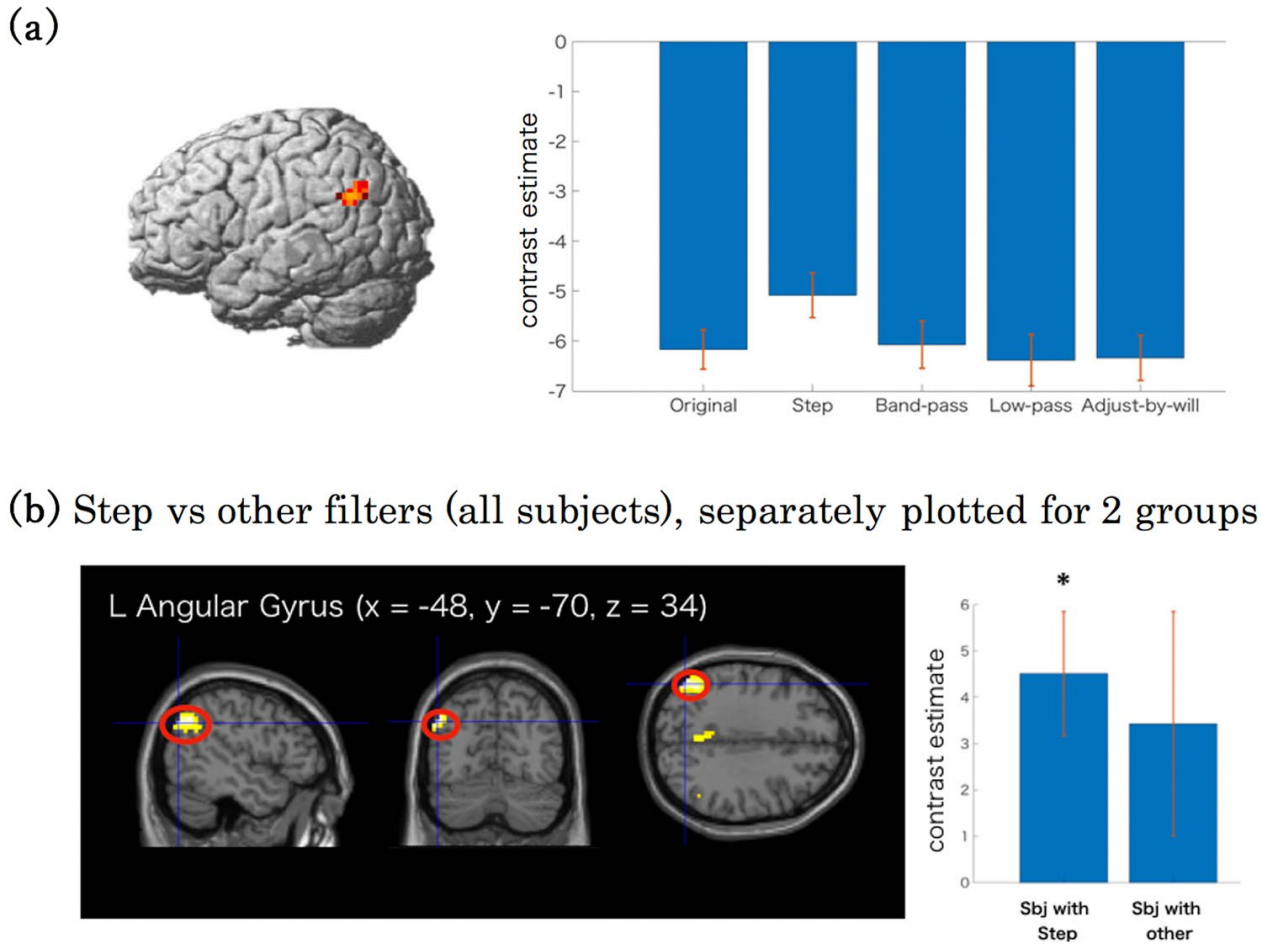
Effects of filter type. (**a**) Left: Significant voxels from ANOVA for the main effect of filter condition, yielding extensive clusters in the left inferior parietal lobes, with peak voxels in the left angular gyrus. Right: Contrast estimate for each filter condition in the left angular gyrus. Displayed contrasts were significant at a voxel-level threshold of *p* < .001, uncorrected. (**b**) Left: Significant voxels for step filtered voice—other contrast. The left angular gyrus showed a contrast peak. Right: Difference in the contrast estimates in the left angular gyrus between the step filter condition and the other filters. Sbj with Step: Subjects who scored high on the step filter. Sbj with other: Subjects who scored high on the other filters. Displayed contrasts were significant at a voxel-level threshold of *p* < .001 combined with an FWE correction of *p* < .05 for cluster size. The error bars represent 90% confidence intervals.

The one-way ANOVA revealed a significant difference in the left angular gyrus (*F*(4,64) = 7.49, *p* < 0.001). In this area, the step filter exhibited less deactivation than the other filters (Fig. 4a).

We then conducted a post hoc analysis, comparing the step filter condition with the other filters using one-sample *t*-tests applied to the voxels that exhibited significant differences in ANOVA with an uncorrected statistical threshold of 0.05. The left panel in Fig. 4b illustrates the differences between the step filter condition and the other filters calculated across all subjects. The step filter induced less deactivations in the left angular gyrus than did the other filters (*t*(16) = 6.09, uncorrected *p* < 0.001 and corrected *p* < 0.05 on cluster level; Table 3, Left panel in Fig. 4b).

**Table 3:** Post hoc analysis of step filter condition > all other filter conditions masked by using the above ANOVA results.

Cluster size	*x, y, z*	*t*	Region
**Step—others**			
125	− 48, − 70, 34	6.09	L Angular Gyrus
	− 48, − 61, 38	5.92	L Angular Gyrus
25	− 3, − 58, 34	4.24	L Precuneus *
	− 6, − 46, 38	3.92	L Precuneus *
18	15, 35, 41	3.69	R Superior Frontal Gyrus *
**Step—others (subjects with step)**			
39	− 48, − 70, 34	5.54	L Angular Gyrus *

Step—Others (the upper part) is the result for all subjects for this contrast, and the lower part is the result for subjects who rated the voice with the step filter to be most similar to the own-voice. Contrasts were significant at a voxel-level threshold of *p* < .001 combined with an FWE correction of *p* < .05 for cluster size. MNI coordinates and *t*-scores are from the peak voxels of a cluster.

*Contrasts were significant at a peak voxel threshold of *p* < .001 (uncorrected), with no cluster thresholding.

To further evaluate the effects of the filters, we separated the subjects into two groups: one of subjects with the highest own-voice score for the step filter, and one of subjects with the highest own-voice score for the other filters. We then contrasted the step filter condition with the other conditions separately for these two groups. In the former group, the left angular gyrus exhibited larger activations with the step filter than the other filters (*t*(12) = 5.54, uncorrected *p* < 0.001; Table 3, Right panel in Fig. 4b). However, such a difference was not observed in the latter group. These results indicate that the activity differences found with the step-filter reflected the tendency that voices with the step-filter were more often perceived as own-voice.

## Discussions

The aim of this study was to investigate the neural basis of voice recognition in humans. We searched for the areas that exhibited lower responses to the voice least similar to the own-voice and the areas that exhibited higher responses to the voice most similar to the own-voice.

In the experiments, we examined the perception of the voices once in a soundproof room and once during the fMRI scans. While the filters perceived as the most and the least similar to own-voice were somewhat consistent across the different environments, we also observed some within-subject variability across the environments. This variability may be attributed to noise, earplugs, modulations applied by noise-cancelling headphones, or posture during the fMRI scans (i.e., subjects needed to lie down during fMRI scans). There was also within-subject variability within the same environment. There was no filter that was always perceived as the most similar to the own-voice. This variability may be due to the task demands; self-recognition by own-face or own-voice stimuli requires a relatively larger cognitive load than other kinds of stimulus processing. Self-recognition tasks have reaction times longer and accuracies lower than discrimination tasks that do not involve self-stimuli^43^. Since all stimuli were generated from the subjects’ own voices in the present study, the rating task might have required a relatively larger cognitive load, thereby reducing within-subject consistency. The filter type rated as the most similar to the own-voice differed across subjects. This individual difference was consistent with our previous study^14^. Such individual differences are reasonable because the bone structures and frequencies of the voices were different across subjects, and the magnitudes of bone and air conduction depend on the sound frequency^44^. We took these within- and between-subject variations into consideration by analyzing the fMRI data based on the subjects’ rating responses, not on the filter types.

The bilateral STG exhibited greater activation for the voice least similar to the subject’s own voice than the voice most similar to their own. The superior temporal area, including the STG, has been reported to show voice-selective responses. The “voice-selective” regions of the STS/STG showed a greater response to vocal sounds than to non-vocal sounds from natural sources or to acoustical controls such as scrambled voices or amplitude-modulated noise^45–47^. Voice recognition involves different aspects of voice processing^48,49^, but it is not yet clear which brain area is involved in each processing. A previous research^39^ examined the neural basis of voice-acoustic processing and voice-identity processing separately by employing the same stimuli for the voice-acoustic categorizing task and for the voice-identity categorizing task. In their experiment, the voices were unfamiliar to all listeners, and listeners were trained to categorize the voice stimuli as a certain person’s voice. Their results revealed that the bilateral middle and posterior STS showed a contrast between trained voice and not-trained voice during the voice-identity task, so these areas were considered to be related to the sensitivity to voice identity. They explained these identity effects through short- and long-term similarity-based mechanisms. With long-term neural sharpening, the stimuli that are more typical in long-term memory elicit reduced neural responses^50,51^.

Our results showed that the bilateral STG and MTG responded less for the voice most similar to the own-voice. This can be interpreted by neural sharpening for the own-voice repeatedly perceived throughout one’s life. In fact, the bilateral temporal areas have been reported to respond differently for the own-voice and for the other’s voices^33,34^, suggesting that these areas respond not only to the large differences such as own vs. others’ voices, but also to the finer variations of the own-voices. The STG and MTG are also involved in the acoustic–phonetic processing, which is necessary to map a stimulus to its phonetic category. A study^42^ reported that medial portions of the bilateral STG and MTG showed increased activation for stimuli that were less prototypical of their phonetic category than for those that were more prototypical. We speculate that each person has an own-voice space where the own-voice is a long-term central representation. When we hear voices that sounds like our own, they were processed in their own voice space, and the typicality-based neural sharpening would occur for their voices. It should be noted that we only used own-voice as familiar stimuli in the present study, but the areas and activations we found may not be specific to the own-voices. Similar results might be observed when we train subjects to be familiar with other acoustic stimuli. However, considering that we have been listening to our own voice throughout our lives, it would be difficult to dissociate the familiarity and the voice to be their own. In fact, neural sharpening can be triggered by any stimuli related to long-term memory. Further studies are required to examine whether these responses are specific to individuals’ own voices.

The bilateral superior temporal area is also known to reduce activation for self-face and self-name stimuli. An fMRI study^24^ compared cortical responses during the recognition of the self-face, self-name, friends, and unfamiliar persons. They observed increased activation in the right temporoparietal regions and the left STS for friends and for unfamiliar persons compared to the self-face and the self-name. These results imply that the bilateral superior temporal area shows domain-general and self-specific characteristics during self-recognition, which may reflect suppression of an automatic preparatory process for social interaction. However, with our experimental design, we cannot conclude whether the responses were domain-general or specific to the voice.

We also evaluated the neural responses using the parametric contrast for the eight rating levels. We did not observe a significant parametric response at the cluster level. However, some contiguous voxels in the bilateral STG respond parametrically to the unfamiliarity of the voice. Our results were insufficient to conclude whether the neural responses to the own voice in the bilateral STG were parametric or categorical. One previous study^52^ measured neural activity in the auditory cortex during overt speech. The subjects received auditory feedback from their own speech. Activation was found to increase in the auditory cortex as the quality of feedback decreased. Their study differs from the present study in two respects. First, they parametrically varied the physical noise level, not the perceptional level. Second, they used the feedback voice as stimuli during self-generated speech. Further studies are needed to examine whether the efferent signal for the speech generation may affect the neural responses to the own-voice.

We also searched for areas that show higher activation to the voices that are most similar to their own, compared to the voices that are least similar to their own (HIGH–LOW). We found no significant clusters. We speculated this is because reducing activations for voices more similar to own voice is more efficient than increasing activations for those voices. Although our subjects did not articulate the stimuli during the experiments, we normally hear our own voices as feedback from articulations. This may reduce the need for processing auditory inputs, as we already know the contents of the sound. This is consistent with a previous study that reported that the auditory cortex showed a reduced BOLD signal in response to vocalization without pitch-shifted feedback compared to pitch-shifted feedback^35^.

Although our main analyses were conducted based on perceptual responses, we also analyzed the effects of the filters applied to the voice stimuli, finding differences in activations in the left inferior parietal lobule, with the peak voxels in the left angular gyrus. This region showed an increase in the BOLD signal in response to the step filter compared to the other filters, resulting from less deactivation of the step-filtered voice. Post-hoc analysis revealed that this particular activation was observed only in subjects whose most own-voice-like filter was the step filter, not in those whose most own-voice-like filter was one of the others. The left angular gyrus is a part of the default network that is deactivated during all goal-directed tasks^53,54^. Thus, deactivation of this area might be related to its function within the default network. The left angular gyrus is also known to be a part of the associative cortex that receives multiple inputs from the modality-specific sensory regions and provides a unique representation of the combined sensory features^55–60^. In addition, the left angular gyrus shows higher activation when the target sentences match propositional prior information than when the target mismatches the prior information^61^. Moreover, the left angular gyrus has the enhanced connectivity with the cerebellum and motor and pre-motor cortical regions, including the supplementary motor area, the pre-central gyrus, and the middle and superior frontal gyri^62^. This parieto-premotor cortical network is involved in the control of attention^63^ and in visual^64,65^, auditory^66^, and cross-modal^67,68^ processing. Thus it is possible that this network acts to direct attention simultaneously to the voice that closely resembles the own-voice.

There are limitations to the present study. First is the repetitive exposure to the own voices in the experimental setting. A study^69^ indicated that the number of exposures to subjects’ own recorded voice affects their own-voice recognition. In our experiments, the subjects were repeatedly presented voices generated from their own voice. Therefore, repetitive exposure to their own voices might have affected their perceptions and neural responses to some degree. Second, the task was to evaluate how the voice sounded like their own. Therefore, we assumed that the neural correlate we observed in this study reflected the subjective experience of recognizing one’s own voice. However, we cannot deny the possibility that the subjects preferred the voices that sounded like their own, and the neural correlate resulting from that preference. In addition, it is unclear whether similar neural correlates can be observed when the subjects passively hear the stimuli. Finally, as we mentioned earlier, the brain activities observed in the present study may not be specific to voice-perception. Similar patterns of activities may be observed with other auditory stimuli with high familiarities. Further studies are required to examine these points.

We conclude that the bilateral temporal area plays a key role in the keen recognition of the own-voice, potentially due to neural sharpening through life-time exposure to their own voices.

## Methods

### Subjects

Nineteen paid volunteers (6 females and 13 males, 21.4 years; *SD* = 2.0; 18–28 years old) who were native Japanese speakers participated in the experiments. All subjects had normal vision and audition. The experiment protocol was approved by the Institutional Review Board at the University of Tokyo, and followed according to Declaration of Helsinki. All subjects provided written informed consent to participate in the study. Two subjects were excluded from data analyses because one missed too many button-presses during fMRI scans and the other one reported a misunderstanding of the task.

### Apparatus and procedure

In Session 1, conducted in a soundproof room, we first recorded each subject's voice. Then, the subject reproduced their “own-voice” by modifying the parameters of their recorded voices, which we named “adjusted-by-will” voice. Each subject’s voice was recorded using a Sennheiser Microphone ME62 (Sennheiser electronic GmbH & Co. KG, Germany) and a Focusrite audio interface (Scarlett 2i4, First Generation model; Focusrite, UK). Audacity, downloaded from www.audacityteam.org, was used to save a digital recording of the voice. All recorded voices were digitized at a 16-bit/44.1 kHz sampling rate. Twenty-six three-syllable Japanese words categorized as neutral^70^ were selected and recorded as stimuli. Each word was presented on a monitor, and the subjects were instructed to articulate the word into the microphone.

After recording all 26 words, the subjects freely modified filters for pitch, vibrato, and frequency features of the original voice (recorded voice) so that the recording sounded like the voice that they heard when they spoke (own-voice). An open-source patch DAVID (Da Amazing Voice Inflection Device^71^) was used to allow subjects to control the auditory features of the voice in real-time. The auditory stimuli were presented through a USB digital-to-analog converter Focusrite audio interface Scarlett 2i4 1st Generation and MDR-XB500 headphones (SONY, Japan). The experimenter instructed the subject how to use the graphical user interface of DAVID step by step until the subject fully understood the procedure. The subjects were allowed to take as much time as they needed until they were convinced that the adjusted voice was their own voice. Vocalization was not restricted while the subject modified the parameters of the voice. Six of the recorded words were used in this voice adjustment procedure, and the remaining 20 recorded words were used later in Sessions 2 and 3; that is, words used in the voice adjustment procedure were not used in the later sessions.

After Session 1, the experimenter created five different types of stimuli by applying filters to the recorded voices. The filters were determined based on previous studies that intended to reproduce the own-voice: + 3 dB for a signal higher than 1 kHz and − 3 dB for a signal lower than 1 kHz as a step filter^11^; a filter passing from 300 to 1200 Hz as a bandpass filter^13^; and a trapezoid-like filter as a low-pass filter^12^. As a result, there were five different types of stimuli: original recorded voice, step filtered voice, bandpass filtered voice, lowpass filtered voice, and adjusted-by-will voice.

In Session 2, conducted in a soundproof room, the subjects rated the voice stimuli on an eight-point scale ranging from *did not sound like their own voice at all* (1) to *sounded very much like their own voice* (8). We called these ratings the “own-voice score.” There were 20 recorded words for each of the five filters. As a result, 100 stimuli were rated once in 100 trials. All 100 trials were randomized within each session. In each trial, a fixation cross was first displayed for 300 ms, followed by a word displayed for 200 ms from the onset of the voice stimulus, whose duration was 800 ms. After the offset of the voice stimulus, a rating scale was displayed. The subjects were asked to respond within 2700 ms by pressing one of eight buttons (Fig. 1b). The subjects were instructed to use all eight buttons during Session 2 so that the responses would not cluster. Figure 4b shows an overview of the experimental design. Subjects were able to take a break after the 50th trial. The visual stimuli were presented on a LCD monitor (BenQ, China) using MATLAB R2015b (The MathWorks, Inc., USA) and the Psychtoolbox (www.psychtoolbox.org). The polarity of the rating scale was reversed after the break. That is, if the least own-voice-like rating was placed on the left side for the first 50 trials, it was placed on the right side for the next 50 trials. The order of the polarity of the rating scale was counterbalanced across subjects.

In Session 3, the subjects conducted the voice rating task while undergoing an fMRI scan. The stimuli and procedures were the same as those in Session 2, except that the stimuli were presented in a rapid event-related design. The order and timing of the stimulus presentation and the inter-trial intervals (ITI) were determined using optseq2 software^72^. The ITI was jittered from 2 to 20 s. During ITI, the subjects fixated on the fixation cross. fMRI data during ITI were used as FIXATION conditions for the analyses. The 20 words used in Session 2 were again used in this session. Each subject participated in 9 or 10 functional scans. Each functional scan comprised 50 trials, yielding 450 or 500 trials in total, with 90 or 100 ratings per filter condition. The polarity of the rating scale was reversed with each functional scan: If the least own-voice-like rating was placed on the left side for the even-numbered scans, it was placed on the right side for odd-numbered ones. The order of polarity was counter-balanced across subjects. After the fMRI scans, we asked the subjects if they pressed the wrong buttons and, if they did, how many times they thought they pressed said buttons. According to these self-reports, their responses were entirely correct or contained few mistakes if there were any. Moreover, we excluded trials in which they missed pressing the buttons.

### MRI/fMRI data acquisition

We employed the same MRI protocol used in our previous study^73^. All MRI images were acquired using a 3 T MRI scanner (Magnetom Prisma, Siemens, Germany) equipped with a 20-channel head coil. Prior to the functional sessions, a high-resolution anatomical image of each subject’s whole brain was acquired using the MPRAGE protocol (TR = 2000 ms, TE = 2.9, TI = 900 ms, flip angle = 9.0°). The slices were aligned parallel to the AC-PC line, and the spatial resolution of the volume was 1.0 × 1.0 × 1.0 mm^3^.

In all functional sessions, BOLD signals were acquired with echo-planar imaging (EPI) sequences. In the experimental sessions, 39 slices aligned parallel to the AC-PC line were acquired in each run to cover the whole brain. The thickness of each slice was 3.5 mm (TR = 2000 ms, TE = 25 ms, flip angle = 75°, in-plane resolution = 3 × 3 mm^2^, FOV = 224 mm, slice gap = 10%). Each functional run comprised 159 volumes. The noise-cancelling headphone (OptoActive, Optoacoustics, Israel) was calibrated during the first 18 s of each functional scan. Trials started after calibration. Auditory stimuli were presented through the aforesaid MR-compatible headphone. The visual stimuli were presented on an MRI-compatible flat-panel LCD display (NNL-LCD, NordicNeuroLab, Norway). Subjects viewed stimuli on the display through an oblique mirror mounted on the head coil. Subjects were instructed to restrain the movements of their heads in order to avoid motion artifacts. The responses at each trial were acquired by two MRI-compatible four-button response devices (Current Designs Inc, USA). Subjects who needed vision correction used plastic correction lenses in the scanner.

### Behavioral data analysis

We compared the own-voice scores under the five filter conditions for each subject to identify the conditions that gave the highest and lowest scores, then examined whether the conditions that gave the highest and lowest scores were consistent between Session 2 (soundproof room) and Session 3 (fMRI).

### MRI data analysis

#### Pre-processing

We employed the same pre-processing protocol used in the previous study^74^. Data processing and analyses were performed using MATLAB (The Math Works, Inc., USA) and SPM8 (Wellcome Department of Cognitive Neurology, UK). The high-resolution structural image was co-registered with the mean image of the EPI series. The structural image was normalized to the Montreal Neurological Institute (MNI) template. All functional images were collected for head motion, and for slice-time, and spatially smoothed with a Gaussian kernel of 8.0 mm (FWHM). The individual volumes were spatially realigned to the mean image by rigid body transformation.

#### General GLM analysis

We applied the general linear model to the fMRI results^26^. A conventional two-level approach for event-related fMRI data was adopted using SPM8. A voxel-by-voxel multiple regression analysis was conducted for the first-level within-subject model. The expected signal changes were modeled for the eight rating conditions. In the model, we also included regressors to account for variance associated with head motion. A voxel-by-voxel statistical inference on the contrasts of the parameter estimates was performed on the second-level between-subject (random effects) model, using one-sample *t*-tests. Activations are reported at a level of significance of *p* < 0.001 uncorrected, and a cluster-level *p* < 0.05 corrected with 10 contiguous voxels. We did not directly measure the acoustic characteristics of our stimuli in our experimental settings. Due to the unusual acoustic environment in the fMRI scanner^75^, we expected the subjects’ perception to differ between the environments (soundproof room and fMRI scanner). We also expected large individual differences, as observed in our previous study^14^. Therefore, we did not have any specific predictions as to which filter would be evaluated as the voice most similar to their own-voice.

## Data Availability

Reported data from all experiments in this study are publicly available on the Open Science Framework (https://osf.io/q34vn/).
